# Prevalence of Clinical Spectrum of Cutaneous Adverse Drug Reactions in Patients Presenting at a Tertiary Care Hospital in Pakistan

**DOI:** 10.7759/cureus.14568

**Published:** 2021-04-19

**Authors:** Ayesha Hina, Sadia Masood, Sajjad Jamil, Saadia Tabassum, Palwasha Jalil, Unzela Ghulam

**Affiliations:** 1 Dermatology, Aga Khan University Hospital, Karachi, PAK; 2 Gastroenterology, Liaquat National Hospital & Medical College, Karachi, PAK; 3 Medicine/Dermatology, Aga Khan University Hospital, Karachi, PAK; 4 Biostatistics and Epidemiology, Aga Khan University Hospital, Karachi, PAK

**Keywords:** cutaneous adverse drug reaction, incidence, age, gender, smoking, socioeconomic status

## Abstract

Introduction: Cutaneous adverse drug reactions (CADRs) are the most common adverse drug reactions reported in the literature. CADRs have resulted in disabling infirmities during hospitalization and complications following outdoor drug therapy. The pattern of CADRs and the responsible drugs usually changes with the introduction of newer drugs and evolving clinical practices. Moreover, several international studies showed variable prevalence, emphasizing the need for local data in light of different socioeconomic and demographic practices. Therefore, the purpose of this study is to evaluate the prevalence of adverse cutaneous drug reactions and identify the clinical spectrum and any potential risk factors.

Methodology: The current study is a descriptive cross-sectional study conducted at Aga Khan University Hospital, Pakistan. One hundred ninety-three patients who met the study inclusion criteria were included. Data were collected from patients on a proforma after taking informed consent. Quantitative data were presented as simple descriptive statistics giving mean and standard deviation, while qualitative variables were presented as frequency and percentages. Effect modifiers were controlled through stratification to highlight the effect of these on the outcome variable. The post-stratification chi-square test was applied and the p-value of ≤0.05 was statistically significant.

Results: A total of 193 patients who had cutaneous adverse drug reactions were included in the study. The mean age in this study was 47.78±8.33 years. One hundred eight (56%) were male and 85 (44%) were female. Out of 193 patients, 135 (69.9%), 50 (25.9%), 24 (12.4%), 12 (6.2%), 20 (10.4%), 11 (5.7%) and six (3.1%) had maculopapular rash, acneiform eruptions, Stevens-Johnson syndrome, erythema multiform, urticaria, fixed drug eruptions and toxic epidermal necrolysis, respectively.

Conclusion: CADRs are a common clinical presentation and awareness and knowledge about their diagnosis and prevention is important. It can be assumed that in our local setup, the clinical trends and medications causing ADRs are strikingly similar to those found in other countries. Physicians commonly come across these cases and they should be well aware of the clinical spectrum of skin reactions to enable early diagnosis and management.

## Introduction

Cutaneous adverse drug reactions (CADRs) are the most common adverse drug reactions reported in the literature [1]. Studies have found the overall incidence of CADRs in developed countries as 1-3%, while the incidence in developing countries is reported to be between 2% to 5% [2]. A wide spectrum of cutaneous manifestations ranging from maculopapular rashes to toxic epidermal necrolysis (TEN) can be caused by different drugs [3]. CADRs have resulted in disabling infirmities during hospitalization and complications following outdoor drug therapy [4]. Certain patient groups seem to be at increased risk of developing such cutaneous drug reactions [5]. The incidence of developing a cutaneous reaction increases with the number of drugs administered [6]. 

Awareness about the culprit drugs can help physicians to choose safer medicines [7]. The Naranjo Scale is a specific questionnaire designed for determining the likelihood of probability and is assigned through a score that is termed as definite, probable, possible, and doubtful [8]. The pattern of drug reactions and the offending drugs show changing trends with the introduction of newer drugs. The reactions can mimic viral exanthems, neoplastic diseases, or collagen vascular diseases [9]. Although these types of cutaneous eruptions are common and complete information about their incidence, severity, complications and ultimate health effects are still unavailable because most of these cases are usually remain unreported [10].

Dermatologists and physicians usually come across many cases of suspected CADRs. Therefore, they should be familiar with different types of skin reactions to enable early diagnosis and prompt withdrawal of the causative drugs to prevent morbidity and mortality [11]. The pattern of CADRs and the responsible drugs usually change with the introduction of newer drugs and evolving clinical practices. Moreover, several international studies showed variable prevalence, emphasizing the need for local data in light of different socioeconomic, demographic, and prescription practices. Therefore, the purpose of this study is to evaluate the prevalence of patients who present with adverse cutaneous drug reactions and also to identify the clinical spectrum and potential risk factors. Currently, there is no local published data available that evaluate these reported cutaneous adverse drug reactions. The incidence and the pattern of the clinical spectrum from this study would give a better understanding of CADRs that would be helpful for local healthcare providers to devise effective management plans for such patients. 

## Materials and methods

**Figure 1:**
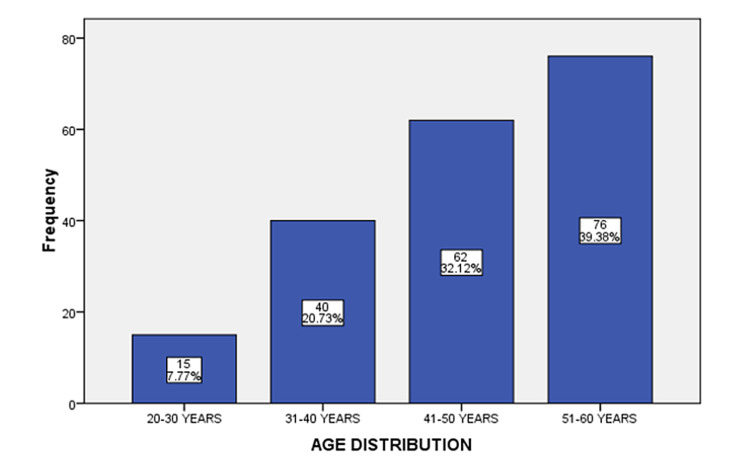
Relation of frequency of cutaneous adverse drug reactions (CADRs) with age. n=193

A descriptive cross-sectional study was conducted at the dermatology outpatient department, Aga Khan University Hospital, Pakistan. The study duration was six months after approval from the ethical review committee (March 2017 to September 2017). The study objectives were to determine the frequency of the clinical spectrum of CADRs and risk factors in patients presenting at a tertiary care hospital, Karachi. The required sample size was 193 using the WHO software, taking the least prevalence of 4.4%, confidence level [CI]=95% and margin of error equal to 2.9%. Patients of all ages and both sexes presenting with cutaneous adverse drug reactions were included. Patients with chronic cutaneous and systemic disorders, using multiple drugs, and who were not willing to participate were excluded. The CADRs were assessed for their causality by performing the Naranjo’s algorithm scale [10], and CADRs were graded as highly probable, possible, and definite according to the scale. Informed consent was obtained from all the participants. A detailed history regarding drug intake, cutaneous eruptions, associated systemic symptoms, the time gap between drug intake and skin eruption, duration, and the indication was recorded. A detailed general physical examination, cutaneous examination regarding morphology, pattern, and distribution of eruption was performed. The findings of quantitative variables (age, reaction time, and duration of cutaneous adverse drug reaction) and qualitative variable (gender, socioeconomic, occupation, marital, educational and smoking status, family history, and duration of cutaneous adverse drug reaction was entered in proforma (Figure 1). As basic descriptive statistics, quantitative data was presented giving mean and standard deviation, and qualitative variables were presented as frequency and percentages. To see the influence of these on the outcome variable, effect modifiers were regulated by stratification. To take a p-value of ≤0.05 as significant, the post-stratification chi-square test was applied.

## Results

**Figure 2:**
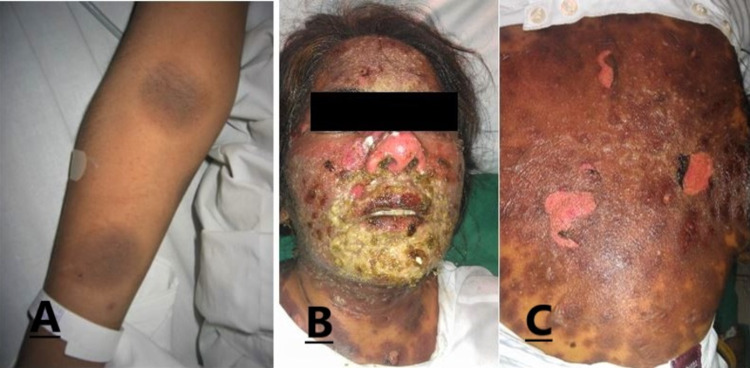
A. Fixed drug eruption on limb. B & C, Toxic epidermal necrolysis showing crusting and peeling of skin

**Figure 3:**
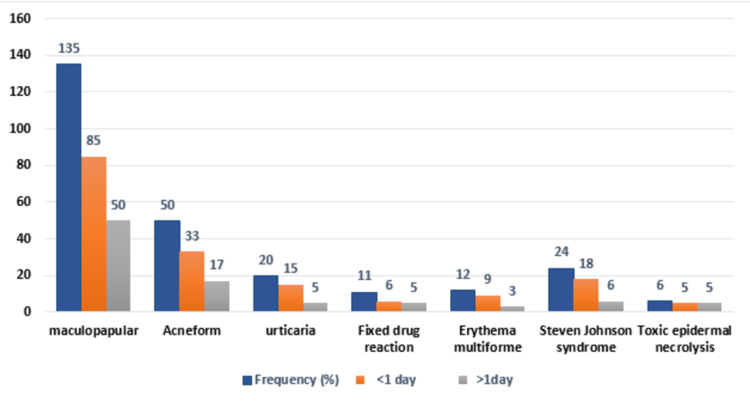
Frequency and Duration of Cutaneous Adverse Drug Reactions

One hundred ninety-three patients were enrolled in the study period in which 108 patients (56%) were male and 85 (44%) were female. The mean age of the patients was 47.78±8.33 years. (Figure 1). The duration of adverse drug reactions between drug ingestion and the onset of eruption ranged from less than a day to more than one day. The maculopapular eruption was found to be the most common reaction observed in 135 (69.9%) patients. Out of those, 85 (63%) patients developed adverse drug reactions in less than 24 hours while 50 (37%) patients developed reactions after 24 hours. Fifty (25.9%) cases were of acneiform eruption, 24 (12.4%) were of Stevens-Johnson syndrome (SJS), 12 (6.2%) cases were of erythema multiform (EM), 20 (10.4%) cases were of urticaria, 11 (5.7%) cases were of fixed drug reaction (FDE) (Figure 2, A) and six (3.1%) were of toxic epidermal necrolysis (TEN) (Figure 2, B&C); 75% of patients with urticaria, EM, and SJS had CADRs in <1 day as compared to FDE which showed the reaction in >1 day in 45.5% of patients. The frequency of CADRs in TEN patients was the same for both as 50% of patients showed adverse drug reaction in <1 day and 50% showed in >1 day (Figure 3).

The prevalence of maculopapular rashes was greater in males (73, 54.1%) than in (62, 45.9%) females but there was no association found between maculopapular rash and gender, age, socioeconomic, occupational, marital, and educational status. The results showed a significant association between erythema multiform and SJS with gender and socio-economic status. Furthermore, a correlation was found between marital status and urticaria, while acneiform eruption had a significant relationship with educational status and family history. No such association was found between selected adverse drug reactions with smoking and age (Table 1). Causality assessment as per the Naranjo algorithm showed that 82 CADRs were probable, 48 were possible, and 63 were definite.

**Table 1:** Socio-demographic representation of cutaneous adverse drug reactions (CADRs) with different variables. n=193 FDE (fixed drug eruption), SJS (Stevens-Johnson Syndrome), TEN (Toxic Epidermal Necrolysis), EM (Erythema Multiforme)

Variables		Maculopapular n (%)	Acne form Eruption n (%)	Urticaria n (%)	FDE n (%)	EM n (%)	SJS n (%)	TEN n (%)
Gender	Male	73 (54.1)	13 (65)	05 (45.5%)	27 (54)	18 (75)	10 (83.3)	03 (50)
	Female	62 (45.9)	07 (35)	06 (54.5)	23 (46)	06 (25)	02 (16.7)	03 (50)
P-value		0.26	0.43	0.26	0.33	0.04	0.03	0.54
Socioeconomic Status	Lower	07 (5.2)	02 (10)	00 (00)	03 (6)	00 (00)	00 (00)	00 (00)
	Lower Middle	14 (10.4)	01 (5)	00 (00)	04 (8)	03 (12.5)	03 (25)	02 (33.3)
	Middle	26 (19.3)	03 (15)	02 (18.2)	08 (16)	00 (00)	00 (00)	00 (00)
	Upper Middle	39 (28.9)	06 (30.6)	04 (36.4)	17 (34)	14 (58.3)	08 (66.7)	02 (33.3)
	Upper	49 (36.3)	08 (40)	05 (45.5)	18 (36)	07 (29.2)	01 (8.3)	02 (33.3)
P-value		0.31	0.90	0.78	0.69	0.00	0.00	0.37
Occupational Status	Employed	99 (73.3)	14 (70)	06 (54.5)	41 (82)	16 (66.7)	09 (75)	05 (83.3)
	Unemployed	36 (26.7)	06 (30)	05 (45.5)	09 (18)	08 (33.3)	03 (25)	01 (16.7)
P-value		0.08	0.02	0.60	0.20	0.48	0.43	0.41
Marital Status	Married	113 (83.7)	20 (100)	09 (81.8)	43 (86)	21 (87.5)	11 (91.7)	06 (100)
	Unmarried	22 (16.3)	00 (00)	02 (18.2)	07 (14)	03 (12.5)	01 (8.3)	00 (00)
P-value		0.51	0.37	0.02	0.57	0.37	0.40	0.33
Educational Status	Illiterate	08 (5.9)	01 (5)	01 (9.1)	01 (2)	01 (4.2)	02 (16.7)	01 (16.7)
	Primary	24 (17.8)	04 (20)	01 (9.1)	06 (12)	04 (16.7)	02 (16.7)	00 (00)
	Secondary	36 (26.7)	02 (10)	04 (36.4)	07 (14)	05 (20.8)	02 (16.7)	01 (16.7)
	Higher	67 (49.6)	13 (65)	05 (45.5)	36 (72)	14 (58.3)	06 (50)	04 (66.7)
P-value		0.25	0.00	0.18	0.89	0.37	0.63	0.40
Smoking Status	Smoker	84 (62.2)	15 (75)	06 (54.5)	33 (66)	18 (75)	09 (75)	03 (50)
	Non-smoker	51 (37.8)	05 (25)	05 (45.5)	17 (34)	06 (25)	03 (25)	03 (50)
P-value		0.23	0.45	0.21	0.34	0.32	0.17	0.36
Family History	Yes	23 (17)	03 (15)	01 (9.1)	12 (24)	05 (20.8)	02 (16.7)	01 (16.7)
	No	112 (83)	17 (85)	10 (90.9)	38 (76)	19 (79.2)	10 (83.3)	05 (83.3)
P-value		0.26	0.04	0.62	0.46	0.58	0.30	0.64

## Discussion

Drug reactions are adverse reactions of the body that occur after the drug administration and are not characteristic of the targeted pharmacodynamics impact. The pattern of drug reactions and offending drugs indicates evolving patterns with the advent of newer drugs. Drug reactions are mostly poorly reported and many queries regarding pathogenesis still have to be addressed. Cutaneous drug reactions are one of the most prevalent forms of drug reaction [12]. In developing countries, the prevalence of CADRs varies from 1% to 3% among patients, whereas some studies reported up to 2% to 5% of the inpatients. However, there is a lack of comprehensive data about the clinical spectrum of drug rashes [13]. The inadequacy of data could be attributed to reasons such as diagnostic dilemmas and lack of awareness and resources. 

In this study, the males were more prevalent with CADRs than females, which was in accordance with two other studies conducted by Suthar and Desai and Sharma et al. [2,14]. Most of the patients were in the range of 51-60 years (39.36%) followed by 41-50 years (32.12%), which is comparable with other research results which established similar observations. Their analysis demonstrates that adults between the ages of 20-49 were at the highest risk of drug reactions [15].

In the current study, the most common CADR was maculopapular rash, which accounted for the overall 69.9%. Garg and John in 2015 had a similar finding where maculopapular rash was the commonest type of CADR (48.8%) [16]. The second most common form of CADR in this study was acneiform eruption (25.91%), although in other studies it was reported in 11.3% of patients, similarly one of the studies also observed this in 1.5% to 7.5% [14-17]. This difference may be attributed to the different ways of drug usage and different feature of an ethnic group. Moreover, one of the studies by Ding and Lee [8] reported 28.1% cases were SJS, 5.7% were TEN, 5.3% were urticaria/angioedema and (5.3%) were FDE, which was similar to our finding. In this study, the time intervals during which manifestations appear (lag time) have been recorded. Maculopapular rash, acneiform, SJS, and urticaria were those patterns observed in the patients within 24 hours. This was comparable with other published studies, which reported the mean reaction time of urticaria was 10 hours followed by 21.70 hours of FDE, the mean lag time for urticaria was 35.27 hours, 3.66 days for TEN, and 3.69 days for SJS respectively. As compared to our results, a study by Agrawal and Ghate showed slightly different results of the lag phase of maculopapular rash, acneiform, and EM which took around 5.66, 10, and 5.7 days [18].

Our results show that there is a significant relationship between EM and SJS with gender and socioeconomic. Some studies have demonstrated similar results [19-20]. One of the implications of this study was to focus on acneiform eruption and occupation, the current study results were statistically significant and few previous studies highlighted a similar significant relationship [21-22]. A positive background of the family history with acneiform eruption can be the predictor of CADR and the current study highlights the same relationship [23]. Moreover, a strong statistical relationship was found between acneiform and education, which shows that inadequate knowledge and the impact of false myths have a significant impact on the development of acneiform eruption [24]. In our study, the majority of the patients were smokers but none of the patterns of CADRs show a possible significant correlation with smoking habits and this trend has also been noticed by Vijendera in his study [25]. As compared to the unmarried population, the married population shows a high prevalence of urticaria, and an association was found between marital status and urticaria. Similar results were reported by Lindegard that they also found a statistical correlation between marital status and urticaria and also their married women show more prevalent to urticaria than un-married [26].

The current study highlighted the prevalence of the clinical spectrum of CADR and more studies need to be conducted in the future to assess the magnitude of CADRs in local setups. Besides, long-term patient monitoring and follow-up could not be done in our study. Despite these limitations, the study shows that health care providers should realize the importance of reporting every drug reaction. Due to the emergence of newer drugs, the patterns of CADRs are changing every year. Physicians should have adequate knowledge of ADRs, especially of newer drugs to minimize such events and manage them effectively.

## Conclusions

In dermatology practice, CADRs are a significant clinical entity, and the severity of these drug reactions usually range from mild maculopapular rashes to fatal eruptions like TEN. Cutaneous drug eruptions are caused by a wide variety of agents and these are among the most frequently reported adverse reactions. These drug reactions are responsible for around 3% of all outdoor and hospitalize patients. Dermatologists and primary care physicians regularly come across such cases and they should be aware of the spectrum of skin reactions to enable early diagnosis, withdrawal of the drugs, and management accordingly. 
